# Postepizootic Persistence of Venezuelan Equine Encephalitis Virus, Venezuela

**DOI:** 10.3201/eid1112.050533

**Published:** 2005-12

**Authors:** Juan-Carlos Navarro, Gladys Medina, Clovis Vasquez, Lark L. Coffey, Eryu Wang, Alexander Suárez, Hernán Biord, Marlene Salas, Scott C. Weaver

**Affiliations:** * Universidad Central de Venezuela, Caracas, Venezuela; † Instituto Nacional de Investigaciones Agropecuarias, Maracay, Venezuela; ‡ Instituto Nacional de Higiene, Caracas, Venezuela; # Sociedad Venezolana de Ciencias Naturales, Venezuela; ¶ Ministerio de Agricultura y Tierras, Barinas, Venezuela; § University of Texas Medical Branch, Galveston, Texas, USA

**Keywords:** encephalitis, alphavirus, pathogenesis, arbovirus, Venezuela, research

## Abstract

Five years after the apparent end of the major 1995 Venezuelan equine encephalitis (VEE) epizootic/epidemic, focal outbreaks of equine encephalitis occurred in Carabobo and Barinas States of western Venezuela. Virus isolates from horses in each location were nearly identical in sequence to 1995 isolates, which suggests natural persistence of subtype IC VEE virus (VEEV) strains in a genetically stable mode. Serologic evidence indicated that additional outbreaks occurred in Barinas State in 2003. Field studies identified known *Culex* (*Melanoconion*) spp. vectors and reservoir hosts of enzootic VEEV but a dearth of typical epidemic vectors. Cattle serosurveys indicated the recent circulation of enzootic VEEV strains, and possibly of epizootic strains. Persistence of VEEV subtype IC strains and infection of horses at the end of the rainy season suggest the possibility of an alternative, cryptic transmission cycle involving survival through the dry season of infected vectors or persistently infected vertebrates.

## Abstract 2

Etiologic subtype IC of virus persists, 5 years after the major 1995 epidemic.

Venezuelan equine encephalitis (VEE) is a reemerging, mosquitoborne viral disease of humans and equines (*1*). Equines serve as highly efficient amplification hosts for mosquitoborne transmission of 2 VEE virus (VEEV) epidemic subtypes, IAB and IC. Humans become infected primarily through the bites of the large numbers of mosquitoes that can be infected by viremic horses. Enzootic VEEV strains of variants ID and IE, closely related antigenically and genetically to each other and to variants IAB and IC, circulate in lowland tropical forests and swamps among small mammals but are incapable of equine amplification to cause epidemics (*2*).

After a long period of inactivity from 1973 to 1992, recent outbreaks in Venezuela (*3**,**4*), Colombia (*5*), and Mexico (*6*) underscore the continued threat of VEE in the Americas. Because recent outbreaks have occurred sporadically, often with many intervening years of epidemic inactivity, the origin of strains of VEEV subtypes IAB and IC was enigmatic for many years. Of several hypotheses proposed (*7*), only 2 have been supported by antigenic and genetic comparisons of VEEV strains and experimental studies. The first hypothesis, suggested by sequence data, is that some epidemics that occurred between the first isolation of VEEV in 1938 and the last outbreak involving a strain of the IAB subtype in 1973 resulted from the use of incompletely inactivated vaccines produced from strains of VEEV subtype IAB (*8*). The lack of VEEV subtype IAB outbreaks since inactivated virus vaccines were replaced by the TC-83 attenuated vaccine strain (a subtype IAB virus) in the early 1970s also supports this hypothesis. The second hypothesis, supported by genetic studies, is that epizootic/epidemic (henceforth called epidemic) subtype IAB and IC VEEV strains arise through mutation from enzootic subtype ID VEEV strains (*9*). The strongest such evidence links a small, 1992-93 Venezuelan VEE outbreak caused by a subtype IC virus strain to sympatric strains of enzootic subtype ID virus (*3**,**10*).

The last major VEE epidemic began in April 1995 in the northern Venezuelan state of Falcon and spread throughout most of northern Venezuela and into La Guajira peninsula of northeastern Colombia to cause ≈75,000–100,000 human cases with ≈300 deaths (Figure 1) (*4**,**5*). Although the total number of equine cases was not reported, it was probably of a similar order of magnitude. The last equine and human cases were reported in December 1995 in Trujillo, Portuguesa, Cojedes, and Guarico States of Venezuela. After the apparent end of the 1995 outbreak, no confirmed epidemic or epizootic VEE occurred in South America for >4 years.

**Figure 1:**
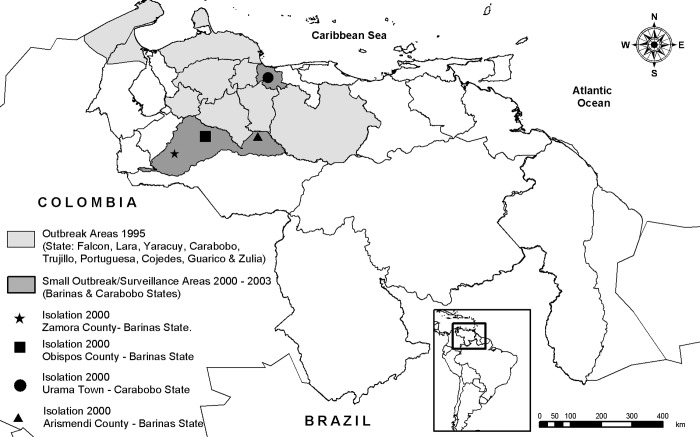
Map of Venezuela showing locations of the 1995 Venezuelan equine encephalitis outbreak and the small outbreaks of 2000 and 2003, along with surveillance study sites.

During December 1999 and February 2000, small, focal outbreaks of equine encephalitis were reported during the end of the rainy season and beginning of the dry season in Carabobo and Barinas States of Venezuela (Figure 1). Similar outbreaks occurred in Barinas State during October 2003. Clinical case descriptions, viral genetic studies, and preliminary surveillance in the region suggested persistence of VEEV subtype IC after 1995 in a cryptic transmission cycle.

## Materials and Methods

### Collection of Animal Samples

Arboviral surveillance was conducted in 2 regions of Barinas State: 1) Zamora and Obispos Counties and 2) Arismendi County (Figure 1). Four farms were studied in Zamora and Obispos Counties from April 2001 to October 2003: 1) El Relámpago (07°57´24´´N, 70°59´20´´W); 2) El Porvenir (07°49´–08°49´N, 70°37´–70°55´W); 3) La Grandeza (07°50´13´´N, 70°44´25´´W), and 4) Boca de Quiu (07°51´–57´´N, 70°43´52´´W). In Arismendi County, studies occurred in October 2003, at the end of the outbreak, on 4 farms: 1) Los Mesones (08°31´52´´N, 68°21´49´´W); 2) Mis Cantares (08°24´09´´N, 68°17´93´´W); 3) Don Eduardo (08°28´36´´N, 68°21´94´´W), and 4) La Espuela (08°28´72´´N, 68°21´68´´W, including equines obtained from El Diamante). These farms, typical of the region, focus mainly on cattle production; equines are maintained for herding.

At each site, Syrian golden hamsters were exposed in *coquito* cages (*11*) for 7 days. Cages were suspended 1.2–1.5 m above the ground in transects at 20- to 25-m intervals, and hamsters were inspected and fed carrots daily. Blood samples were collected by cardiac puncture from moribund hamsters and from those surviving exposure; heart and spleen samples were then dissected and preserved in liquid nitrogen. The maintenance and care of animals complied with guidelines of the University of Texas Medical Branch and the Instituto Nacional de Higiene.

Parallel to the hamster transects, Sherman and Tomahawk traps were used for collecting small mammals, as described previously (*12*). Animals were bled by cardiac puncture and identified by using taxonomic keys (*13**,**14*).

### Mosquito Collections

Mosquitoes were collected with CDC light traps (*15*) baited with dry ice and suspended ≈1.5 m above the ground. Mosquitoes were identified by using taxonomic keys (*16**–**19*) and reference collections (*20*).

### Virus Isolations and Identification

Sera and tissues from equines, sentinel hamsters, and wild rodents were injected into 1- to 3-day-old mice or African green monkey kidney (Vero) cells for virus isolation. Viruses indicated by mouse deaths or cytopathic effects in cell culture were identified by hemagglutination inhibition (HI) tests. Subtypes of the VEEV isolates were determined by immunofluorescence by using monoclonal antibodies (MAbs), as described previously (*21*), and by amplifying by reverse transcriptase–polymerase chain reactions (RT-PCR) a portion of the PE2 envelope glycoprotein precursor gene, followed by sequencing and phylogenetic analyses, as described previously (*22*).

### Genetic Analyses of Virus Isolates

The complete genomes or partial PE2 envelope glycoprotein precursor sequences of VEEV strains were amplified by RT-PCR using Superscript reverse transcriptase (BRL, Bethesda, MD, USA) and Pfu polymerase (Stratagene, La Jolla, CA, USA), as described previously (*23*). After electrophoresis, amplicons were extracted from 1% agarose gels and sequenced directly by using previously described primers (*23*) and the Applied Biosystems (Foster City, CA, USA) Prism automated DNA sequencing kit to produce consensus sequences. Genomic sequences, excluding the 5´ terminal 20 nucleotides (nt) derived from the primer, or PE2 sequences, were submitted to the GenBank library under accession numbers AY973944 and AY986475. Sequences were aligned with homologous VEEV sequences in the GenBank library and analyzed by using neighbor-joining and maximum parsimony methods implemented in the PAUP 4.0 software package (*24*) and Bayesian methods with MrBayes 3 (*25*).

### Antibody Detection and Characterization

Serum samples were screened for VEEV antibodies by using HI with antigens prepared from the TC-83 attenuated virus vaccine strain and the South American eastern equine encephalitis virus strain C-49 (*26*). Antibodies from serum samples with titers >1:20 were confirmed by using 80% plaque reduction neutralization tests (PRNTs). To determine the VEEV subtypes that produced reactive sera, a blocking enzyme-linked immunosorbent assay (ELISA) with purified VEEV antigens and enzootic- or epidemic-specific MAbs (*21*) was used, as described previously (*27*). Negative control serum specimens were obtained from animals in non-disease-endemic locations with no history of VEE.

## Results

### Description of Outbreaks

In February 2000, the Venezuelan Animal Health Service received a report of an equine encephalitis outbreak, consistent with VEE, on the Bella Vista farm in the Curito Abajo area of Zamora County, Barinas State. Simultaneously, another outbreak occurred on the Los Cerros farm in Obispos County (Figure 1). Five serum specimens were collected from stablemates of affected horses on the Bella Vista farm; 2 yielded VEEV isolates. One brain sample collected at necropsy during April 2000 on the Los Cerros farm yielded VEEV. Suspected VEE was reported in Obispos, Zamora, Pedraza, and Miranda municipalities before these VEEV isolates were confirmed at the Instituto Nacional de Investigaciones Agropecuarias in April 2000. In addition, retrospective examination of epidemiologic records showed suspected VEE cases in Zamora County (Curito Abajo) since December 1999.

After the February 2000 outbreaks, 8 additional foci with 35 fatal equine cases were reported: 3 outbreaks in Zamora (15 deaths), 2 in Obispos (13 deaths), 2 in Pedraza (7 deaths), and 1 (no deaths) in Sucre County. Regional equine vaccination coverage with the strain TC-83 attenuated virus vaccine was 24% before the outbreaks, and additional vaccination initiated around all apparent foci in early 2000 increased coverage to 51%.

A geographically distant equine case consistent with VEE occurred in April 2000 in Carabobo State (Figure 1). The affected horse had been moved from south Guarico State (borders Obispos County of Barinas State) 10 days before onset of disease. Virus was isolated from its brain after euthanization and from the serum of a recently vaccinated stablemate. Another equine death had occurred on the same farm, but the horse had been incinerated before the cause of death could be confirmed.

Another outbreak consistent with VEE was reported in October 2003 in northeastern Barinas State around the town of Arismendi and along the nearby Guanare River basin (Figure 1). Because the affected farms were difficult to access and farmers did not initially report the cases to avoid equine quarantines, samples from affected horses were not obtained.

### Equine Serology

A total of 619 equine serum specimens from 8 different municipalities in Barinas State were tested for VEEV and eastern equine encephalitis virus (EEEV) antibodies by using HI (Table 1). Zamora and Pedraza municipalities showed the highest rates of VEEV seropositivity (44% and 48%, respectively); although vaccination was ongoing at the time of sampling, and some seropositivity undoubtedly resulted from vaccination, vaccine coverage of only ≈24% suggested that some horses were naturally infected. The blocking ELISA (*27*) cannot distinguish between strain TC-83 and subtype IC VEEV infections, so the VEEV strain origins of equine antibodies could not be determined.

**Table 1:** Results of hemagglutination inhibition assays to detect Venezuelan equine encephalitis virus antibodies in horses

Municipality	No. serum samples tested	No. positive (%)
Obispos	199	46 (23)
Zamora	128	61 (48)
Bolivar	18	0
Pedraza	23	10 (44)
Barinas	172	37 (22)
Arismendi	9	0
Sucre	65	1 (2)
A. Arvelo Torrealba	5	1 (20)
Total	619	174 (28)

### Surveillance

To investigate circulation of VEEV, small mammals, known to be reservoir hosts of VEEV, and mosquitoes were trapped at 4 sites near the equine cases. Bovines <2 years of age that lived on the same farm for their entire life were bled as sentinels to detect recent VEEV circulation. The region of the first outbreak in 2000 included a part of Zamora and Obispos Counties, formerly included in the Ticoporo Forest Reserve (previously a rainforest, 8°12´N/70°56´W) between the Quiu and Michay Rivers, on the border between the western Llanos (plains and savannas) and the southern Andes Mountains. This area has been deforested for timber, cattle ranching, and crop farming, leaving only forest fragments and gallery forests with slopes of <1% grade and an altitude of ≈200 m. The forested area decreased from 186,000 hectares (ha) in 1955 to 5,000 ha in 2002 (http://www.tierramerica.net/2002/1124/ecobreves.shtml).

The Barinas State rainy season is from April to December, and the dry season is from January to March, with mean temperatures of 22°C to 30°C. Annual precipitation averages 1,729–1,995 mm. The flora are typical of the Llanos and lowland Andean Mountains, including *Ceiba pentadra*, *Bombacopsis quinata*, *Spondia mombin*, *Chrysophyllum sericeum*, *Pouteria anibaefolia*, *Guazuma tomentosa*, *Attalea maracaibensis*, and *Roystonea venezuela*. During the end of the rainy season, rivers flood adjacent lowland forests and generate habitat for water lettuce (*Pistia stratiotes*), a floating plant used as a breeding site by some *Culex* (*Melanoconion*) mosquito species.

The region of the second VEE outbreak in 2000 (Arismendi County) also comprises lowland savannas in the Guanare River basin and includes extensive cattle production and fragmented forests resulting from deforestation. This part of Barinas State borders Guarico, Cojedes, and Portuguesa States to the north and northwest, and Apure State to the south (Figure 1). The flora is characteristic of lowland Llanos (95-m altitude) and flooded savannas: *Copernicia tectorum* (Llanos palms), *Hymenachne amplexicaulis*, *Leersia hexandra*, and *Luziola spruceana*. The climate is similar to that described above.

### Mammal Collections

A total of 130 small mammals were collected during 6,600 trap-nights, for a success rate of 1.9%. The captures and serologic data are shown in Table 2. The spiny rat (*Proechimys guairae*) and the cotton rat (*Sigmodon hispidus*), both belonging to known enzootic VEEV reservoir genera (*12**,**28**,**29*), as well as the cane mouse (*Zygodontomys brevicauda*), were abundant at most sites. No virus was isolated from wild mammals; HI antibodies to VEEV were detected in 4 (6.8%) of 58 *P. guairae*, 1 (25%) of 4 *Didelphis marsupialis*, and 1 (6.6%) of 15 *S. hispidus*. However, HI titers were low (<1:20), and none was confirmed by PRNT (<20). No EEEV-reactive antibodies were detected. These results suggest either very low VEEV antibody titers in some rodents or nonspecific HI reactivity.

**Table 2:** Serologic results from equines, bovines, and wild mammals in regions of the 2000 and 2003 VEEV outbreaks*

Location	Dates of collection	Species or common name	No. collected	Fraction HI seropositive	Fraction of HI-positive samples positive by PRNT	PRNT titers
Obispos	May 2002	*Proechimys guairae*	10	0/10	NT	NT
		*Zygodontomys brevicauda*	47	0/47	NT	NT
Zamora	May 2003	*Akodon urichi*	2	0/2	NT	NT
		*Oryzomys talamancae*	1	0/1	NT	NT
		*Sigmodon hispidus*	13	0/13	NT	NT
		*Zygodontomys brevicauda*	1	0/1	NT	NT
		*Didelphis marsupialis*	2	0/2	NT	NT
		*Proechimys guairae*	18	0/18	NT	NT
		*Rattus rattus*	2	0/2	NT	NT
		Bovines	4	0/4	NT	NT
	June 2003	*Proechimys guairae*	6	1/6	0/1	<20
	Sep 2003	*Didelphis marsupialis*	1	1/1	0/1	<20
		*Proechimys guairae*	11	4/11	0/4	<20
		*Sigmodon hispidus*	1	1/1	0/1	<20
		Bovines	20	0/20	NT	NT
	Nov 2003	*Proechimys guairae*	10	0/10	NT	NT
		*Sigmodon hispidus*	1	1/1	NT	NT
Arismendi	Oct–Nov 2003	Bovines	48	12/48	8/12	40–640
Antonio Jose de Sucre/Zamora	June 2003	*Proechimys guairae*	3	0/3	NT	NT
		*Didelphis marsupialis*	1	0/1	NT	NT
Totals		Wild mammals	130	7/130 (5%)	0/7	<20
		Bovines	72	12/72 (17%)	8/12	40–640

*VEEV, Venezuelan equine encephalitis virus; HI, hemagglutination inhibition; PRNT, plaque reduction neutralization test; NT, not tested.

### Bovine Serology

Bovines are effective VEE sentinels because they are naturally infected and seroconvert but no disease develops and they are not vaccinated (*30*). We bled cattle <2 years of age that had resided on the same farm for their entire life. The Zamora site showed no evidence of bovine seropositivity, with 0 of 4 positive for VEEV antibodies in May and 0 of 20 in September 2003 (Table 2). However, the Arismendi site had a 25% bovine VEEV seropositivity rate (12/48) from October to November 2003, and all positive serum samples were negative for EEEV. To determine whether the seropositive cattle were infected by enzootic or epidemic (subtype IC) VEEV strains, we used a blocking ELISA that distinguishes antibodies based on their ability to block the reaction of subtype-specific monoclonal antibodies (*27*). Five of the 8 PRNT-positive bovine serum specimens had consistently higher blocking activity against the enzootic virus-specific MAb, indicating exposure to enzootic VEE-complex alphaviruses (Table 3). However, 2 samples (166, 167) had similar blocking activities against both enzootic and epidemic virus–specific MAbs, which suggests either infection with both enzootic and epidemic VEEV phenotypes or nonspecific reactivity against 1 subtype.

**Table 3:** Results of the blocking ELISA to determine VEEV subtypes producing seroconversion in HI- and PRNT-positive bovines from Arismendi municipality, Barinas State, Venezuela*

Sample no.†	% inhibition of epizootic–specific MAb 1A3A–5 binding at indicated serum dilution			% inhibition of enzootic–specific MAb 1A1B–9 binding at indicated serum dilution		
	1:4	1:12	1:36	1:4	1:12	1:36
156	3.0	8.5	–5.0	52.4	17.8	–34.3
158	12.4	13.9	–3.6	27.0	–20.4	–37.0
166	35.6	12.5	6.2	30.7	42.7	–21.0
167	59.6	42.0	3.7	65.7	17.1	–9.9
172	–11.7	–15.4	–13.8	37.5	32.1	–19.0
175	8.2	5.0	2.1	48.5	21.5	–20.0
178	23.1	15.1	2.2	69.7	44.9	–5.9
204	–1.1	–10.8	–22.4	75.8	56.4	29.7
Negative control†	4.1	6.6	5.4	15.2	23.1	–5.7
Negative control†	9.4	14.1	12.0	8.0	14.5	15.6
Negative control†	5.8	11.4	11.3	17.5	23.2	11.0
Positive control†	18.8	9.5	–8.0	65.3	51.0	29.0
Positive control†	16.4	1.4	1.7	50.1	16.7	18.9

*ELISA, enzyme-linked immunosorbent assay; VEEV, Venezuelan equine encephalitis virus; HI, hemagglutination-inhibition; PRNT, plaque reduction neutralization test; MAb, monoclonal antibody.

†Negative controls are bovine sera from locations with no history of VEE; positive controls are human sera from virus isolation–confirmed, subtype ID VEEV infections (note that the assay is species–independent).

### Mosquito Collections

Three CO_2_-baited CDC light traps were stationed for 2 consecutive days on each farm to assess possible VEEV vectors as follows: May, June, and September–October, 2003 (Zamora County); October–November 2003 (Arismendi); and December 2003 (Obispos). These months correspond to the dry season and the rainy season. Maximum catches were obtained in December 2002 and November 2003, at the end of the rainy season. A total of 21 mosquito species and 5 unidentified taxa (to species level) were collected (Table 4). The most abundant species captured were *Cx*. (*Melanoconion*) *dunni*, *Mansonia titillans*, *Cx*. (*Mel*.) *spissipes*, *Coquillettidia aribalzagae*, *Ae. scapularis*, *Cx.* (*Mel*.) *aikeni* sensu lato (*ocossa* and *panocossa*), and *Psorophora albipes*. Also, a large number of unknown *Cx*. (*Mel*.) spp. belonging to the Melanoconion Section were captured. No viruses were isolated from mosquitoes; however, 2 of the most abundant species, *Cx. ocossa* and *Cx. panocossa*, have been incriminated as enzootic VEEV vectors and *Ma. titillans* and *Ae. scapularis* were implicated as potential bridge vectors that may export VEEV from sylvatic, enzootic foci in Venezuela (*31*). However, typical epidemic vectors such as *Ae. taeniorhynchus* and *Ps. confinnis* were not present.

**Table 4:** Mosquitoes collected in the regions affected by the 2000 and 2003 Venezuelan equine encephalitis outbreaks

Species	Study site			Total
	Zamora	Obispos	Arismendi	
*Culex* (*Melanoconion*) *dunni*	133	924	25	1,082
*Cx*. (*Mel*.) *spissipes*	26	15		41
*Cx*. (*Mel*.) pedroi*	1			1
*Cx*. (*Mel*.) *theobaldi*	1			1
*Cx*. (*Mel*.) ocossa*		13	6	19
*Cx*. (*Mel*.) panocossa*		5		5
*Cx*. (*Mel*.) sp. Mel Section	24	120		144
*Cx*. (*Cux*.) spp.	800	132	45	977
*Psorophora albipes*	39	18		57
*Ps. ferox*	2	5		7
*Ps. lineata*	1			1
*Ps. cingulata*	7	3		10
*Aedes fulvus†*	2			2
*Ae. scapularis†*	6	44	9	59
*Uranotaenia calosomata*	17	5		22
*Trichoprosopon digitatum*	12	1		13
*Trichoprosopon* sp.		1		1
*Wyeomyia* (*Phoniomyia*) sp.	2			2
*Uranotaenia geometrica*	2			2
*Coquillettidia arribalzagae*	6		70	76
*C. nigricans*			5	5
*C. juxtamansonia*		7		7
*Mansonia titillans*		10	169	179
*Limatus asulleptus*		4		4
*Aedomyia squamipennis*			3	3
*Anopheles* sp.			2	2

*Proven enzootic vectors of Venezuelan equine encephalitis virus.

†Suspected bridge vectors.

### Isolation and Genetic Analyses of VEEV Strains from Equines

Two equine brain specimens and 3 serum samples from stablemates yielded mouse deaths with CPE-inducing activity in brains. Antigenic analyses that used MAbs indicated that all isolates belonged to subtypes IAB/C. Four sequences of RT-PCR amplicons covering the PE2 gene were identical to the subtype IC strains 6119 and 3908 from the 1995 epidemic, and also to the subtype IC strains P676 and V198 from the 1962–64 epidemic (Table 5) (*23*). The exception was strain 254818, isolated from a stablemate of a deceased horse in Carabobo State after strain TC-83 vaccination was initiated; this strain had a PE2 sequence identical to that of strain TC-83 (*32*), with the exception of a single nucleotide difference at genome position 8845 that encoded a Lys to Met change at E2 amino acid position 115.

**Table 5:** Sequence comparisons between the year 2000 VEEV virus equine isolates and those from the 1995 epidemic*

Virus strain†	State of isolation	Horse sample	Date of collection	Passage history	Comparisons to 1995 strain 6119		Comparisons to 1995 strain 3908	
					nt differences‡	aa differences§	nt differences¶	aa differences#
254934	Barinas	Brain	Apr 10, 2000	sm-1	C1248T	–	A1443G	–
					G6325A	nsP4/V208I	T4975C	
					G9165A	E2/E199K	C5292T	–
					T10913C	–	T5475C	–
					C11237T	–	G6325A	nsP4/V208I
							A6498T	–
							G9159A	E2/E199K
							C11237T	–
255010	Barinas	Serum	Feb 20, 2000	sm-2	C1248T	–	A1443G	–
					T10913C	–	T4975C	–
					C11237T	–	C5292T	–
							T5475C	–
							A6498T	–
							G9159A	E2/E199K
							C11237T	–
255005*	Barinas	Serum	Feb 20, 2000	sm-3	0	–	0	–
255057*	Carabobo	Brain	April, 2000	sm-1, V-1	0	–	0	–

*VEEV, Venezuelan equine encephalitis virus; nt, nucleotide; aa, amino acid.

†Only partial PE2 envelope glycoprotein precursor gene sequences determined (817 nucleotides).

‡Strain 6119 residue is indicated first, followed by genome position and year 2000 strain residue.

§Strain 6119 residue is indicated first, followed by protein and amino acid position and year 2000 strain residue.

¶Strain 3908 residue is indicated first, followed by genome position and year 2000 strain residue.

#Strain 3908 residue is indicated first, followed by protein and amino acid position and year 2000 strain residue.

To increase phylogenetic resolution, the complete genomes (excluding the 5´ terminal 20 nt that incorporated PCR primers into amplicons) of representative strains from Carabobo (255010) and Barinas States (254934) were sequenced. The most closely related sequence to both isolates was strain 6119, isolated in May, soon after the beginning of the 1995 VEE epidemic in Falcon State. This strain differed from strains 255010 and 254934 by only 1 and 4 nt, respectively. Slightly more distantly related was strain 3908 from Zulia State in September 1995, followed by strains from the 1962–64 Venezuelan/Colombian epidemic. Only 1-nt difference among the year 2000 and 1995 VEEV isolates encoded an amino acid difference; strain 254934 had Lys at E2 position 199, whereas all other strains had Glu (Table 5).

Phylogenetic analyses that used all methods indicated that strain 6119 had a sequence identical to the predicted ancestor of strains 255010 and 254934, and branch lengths indicated interepidemic evolutionary rates of 1.7–7.0 × 10^–5^ substitutions/nucleotide/year. In contrast, during the 1995 outbreak, relative branch lengths of strains 6119 and 3908 indicated a faster evolutionary rate of 2.0 × 10^–4^ substitutions/nucleotide/year. Relative rate analyses of the 1962–64 epidemic clade resulted in similar estimates of intraepidemic evolution from 2.2–4.4 × 10^–4^ substitutions/nucleotide/year, similar to estimates of ≈3 × 10^–4^ substitutions/nucleotide/year for enzootic VEEV in Venezuela (*23*). These data indicate that the subtype IC VEEV strains persisted in Venezuela from 1995 to 2000 in a genetically stable manner, with ca. 10-fold slower rates of nucleotide substitution than are estimated to occur during epidemic or enzootic circulation.

## Discussion

Of 5 major hypotheses proposed to explain the source(s) of strains of subtypes IAB and IC responsible for all major VEE outbreaks (*7*), 2 are supported by previous studies: 1) several of the later VEE outbreaks caused by subtype IAB strains were probably initiated by the use of incompletely inactivated vaccines produced from early, wild-type, equine-virulent isolates (*8**,**33*); and 2) all subtype IAB and IC strains evolved independently from an enzootic lineage of subtype ID VEEV that circulates in western Venezuela, Colombia, and northern Peru (*2**,**22*). Johnson and Martin (*7*) also hypothesized that epidemic strains might persist between outbreaks in cryptic transmission cycles that have been overlooked, despite postepidemic surveillance in the affected areas of Colombia (*11**,**12*) and Venezuela (*12**,**34**,**35*).

We report the first direct evidence that supports postepidemic circulation of epidemic VEEV. During 2000 in western Venezuela, 5 years after the apparent end of the 1995 epidemic, 4 isolates of VEEV nearly identical to 1995 strains were associated with equine encephalitis in Barinas and Carabobo States. Viral sequences had undergone virtually no evolutionary change during the interepidemic period, in contrast to epidemic and enzootic virus circulation, in which a relatively steady rate of nucleotide substitutions, on the order of 2–4 × 10^–4^ substitutions/nucleotide/year, occurs (*23**,**36*). From 1995 to 2000, the subtype IC strain underwent an ≈10-times slower evolution, which suggests less replication than normally occurs in rodent reservoir or equine amplification hosts and mosquitoes during horizontal transmission.

Our seroprevalence data from bovines also suggest that enzootic VEEV strains may have been circulating in the affected regions. Although we did not identify bovine serum that exhibited blocking activity solely against the epidemic virus–specific MAb, some samples reacted in both epidemic- and enzootic virus-specific ways. Larger samples of bovine and rodent serum are needed to more conclusively assess the subtype(s) of VEEV strains circulating in the region.

Although we could not identify the critical reservoir hosts and vectors that allow VEEV to persist in Barinas State, the occurrence of equine cases at the end of the rainy season and beginning of the dry season suggests fundamental differences from normal epidemic or enzootic circulation. Epidemic VEE generally occurs during the peak of the rainy season, when floodwater mosquitoes are abundant. Although mosquito surveillance was not conducted during the Barinas or Carabobo outbreaks, collections during the same season in 2001 indicated relatively small populations on the affected farms. Although our more recent mosquito collections included known enzootic VEEV vectors (Table 4), these mosquitoes are not known to transmit epidemic virus strains, and we did not detect VEEV antibodies in the rodents with which they are typically associated in sylvatic, enzootic foci. Typical epidemic vectors were not abundant in the affected regions. These results suggest the possibility that more xerophilic vectors other than mosquitoes might have been responsible for subtype IC VEEV maintenance and transmission to horses.

Ticks are susceptible to experimental infection by VEEV, although rates of oral and transtadial transmission tend to be low (*37**–**39*), and persistence for up to 171 days has been demonstrated. The effect of persistent tick infection on alphavirus genome stability has not been evaluated, but alphavirus infection of mosquitoes involves early replication for ≈1 week, followed by declining replication due to poorly understood modulating factors that probably include RNA interference (*40*). If similar mechanisms occur in infected ticks, long-term persistence could result in the levels of genetic stasis we observed in subtype IC VEEV from 1995 to 2000. To evaluate this hypothesis, more extensive surveillance designed to identify the vector(s) and reservoir host(s) in Barinas State is ongoing.

**Figure 2:**
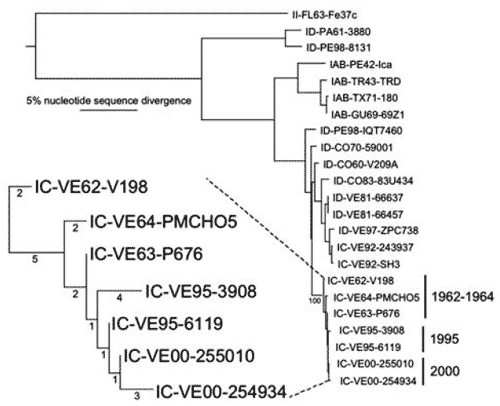
Phylogenetic tree generated from maximum parsimony analysis of genomic sequences of Venezuelan equine encephalitis virus (VEEV) strains 255010 and 254934 and homologous GenBank sequences from the 1962–64 and 1995 VEEV outbreaks, as well as other representative VEE complex alphavirus strains. Numbers indicate bootstrap values for groupings to the right. Enlargement on the lower left shows the 1962–64 and 1995–2000 clades, with numbers indicating nucleotide substitutions accompanying VEEV evolution.

Our results also call into question previous estimates of alphavirus evolutionary rates that suggested a laboratory source for the 1995 Venezuelan epidemic. The inconsistency between the genetic stasis observed in subtype IC strains isolated from 1962–1964 versus 1995, and rates of nucleotide substitution observed during enzootic or epidemic VEEV circulation, suggested a laboratory source for the 1995 outbreak (*23*). The common use in Venezuela of antigens prepared from a 1963 strain (P676), from which active virus was isolated, and its similarity to the predicted progenitor of the 1995 outbreak also supported this hypothesis of a laboratory origin. We consider it highly unlikely that the year 2000 Carabobo and Barinas outbreaks resulted from laboratory strains because of the following factors: 1) in Venezuela, wildtype epidemic VEEV strains have largely been replaced for antigen preparation by the strain TC-83 vaccine virus to minimize the possibility of a laboratory-initiated outbreak; 2) the 2000 isolates do not group phylogenetically with strain P676 as the 1995 strains do (Figure 2); and 3) unlike Falcon state, where the 1995 outbreak began, the locations of the 2000 outbreaks are far from the diagnostic and vaccine production laboratories that work with VEEV. Also, the Instituto Nacional de Investigaciones Agropecuarias, where the year 2000 VEEV strains were isolated, had not worked with subtype IC VEEV for many months. Furthermore, the isolation of the strain TC-83 virus from a stablemate of the encephalitic horse at the Carabobo site during vaccination efforts argues against any laboratory contamination with a subtype IC strain. This evidence strongly suggests that the 2000 outbreaks involved naturally circulating VEEV strains that were maintained in a genetically stable state since 1995. Thus, based on genetic stasis and other factors, the previous conclusion that the 1995 outbreak may have had a laboratory origin, should be reevaluated. Since epidemic strains of VEEV can be maintained for at least 5 (1995–2000 or 2003) and possibly even 31 (1964–1995) years between epidemics, equine vaccination efforts and surveillance should be implemented continuously in Venezuela and Colombia.
